# Dental Responsibility of Correct Oral Diagnosis*Read before the Reunion meeting of the National Alumni Association of the Baltimore College of Dental Surgery, Baltimore, Md., May 30 to June 1, 1922.

**Published:** 1923-02

**Authors:** B. Lucien Brun

**Affiliations:** Baltimore, Md.


### DENTAL RESPONSIBILITY OF CORRECT ORAL DIAGNOSIS*

BY B. LUCIEN BRUN, D. D. S., BALTIMORE, MD.

The importance of correct oral diagnosis has been impressed upon you; may we proceed a step further and see the great importance and realize the responsibility that rests upon us as dentists in correctly applying these principles?

Modern medicine in the recognition of the role played by focal infection, has marked an advance far-reaching; and this has been closely followed by the important discovery that septic lesions of the mouth may be foci for many acute or chronic diseases of systemic nature. This

---

* Read before the Reunion meeting of the National Alumni Association of the Baltimore College of Dental Surgery, Baltimore, Md., May 30 to June 1, 1922.

in itself has been greatly responsible for the change in the relationship between the physician and the dentist.

· Within the last decade much has been written, much clinical evidence has been collected by dentists, to prove that in certain instances and under certain conditions gingival and periapical infections are responsible for general disease. The broad range of the literature which is available on this subject by such men as Billings, Irons, Grieves, Rosenow, Rhein, Hartzell, Henrici, and others, is familiar to many of us. We must admit that there has been a great loss of valuable time in co-ordinating the findings of these men, both medical and dental, who have so earnestly striven by research and clinical effort to throw light upon this subject of oral sepsis and its relation to systemic infection.

It is conceded today that a complete examination of the patient by different specialists, trained as diagnosticians in their particular branch of medicine, and the correlated findings of these studied by the internist, who can then the more intelligently compare their relation to the general disease and determine the treatment, is the ideal course of procedure.

Such a diagnostic study is not, however, indicated in all cases. This course is decided by the internist after his preliminary study of the patient has determined whether or not focal infection is to be included as a possible causative factor.

"Infection plays no part in many diseases, while others are infectious without visible foci; others again are focal in origin; or again focal infections may lower resistance, as a secondary, but important, factor in all of the groups."

The necessity of an understanding of the pathology of the mouth lesions and proficiency in making a dental diagnosis are of prime importance, that a comprehensive report can be made to the physician for his guidance; and that the dentist, by his knowledge, will be able to check against his own diagnosis, should he be called upon to correct the oral condition, by either such treatment as might be indicated or the more radical steps of surgical procedure. A knowledge of dento-histo-pathology, in a broader sense must be acquired, for from the medico-dental standpoint we do not, at the present time, speak the same language.

Teeth formerly were looked upon as organs entirely apart from the rest of the body; they are now included with the important atria through which disease may enter, and it is along these lines that we must consider the relation of dental infection to general health. This cannot be done if we ignore the discussion of focal infection in general.

That there are many sites where chronic infection may occur is well established; and the mere mention of this will the more emphasize the necessity of a broader relation between the physician and the dentist.

General disease which is caused either directly or indirectly by dental foci of infection, is our present consideration. The portals of entrance are by septic invasion either directly through the pulp canal, or *via* the gingival crevice, both avenues of attack may result in periapical disease.

Of teeth, with vital pulps; or sound teeth, with pulps diseased by pyorrhea; abraded or eroded teeth, with occasional pulpal symptoms; or average teeth, with pulps diseased through trauma, senility or surgical exposure; or, of carious teeth, with infected pulps, Grieves says:

"If pulps can be removed under as nearly aseptic conditions as possible by careful chemico-mechanical instrumentation, leaving only inaccessible vessels and vital pulp-shreds in the immediate foraminal openings of a vital apex, which has not been cauterized or over sterilized; or, if these strong agents can be used without their passing out into the membrane, if all this can be accomplished without perforation and encapsulation, avoiding infection from debris, then any canal-filling method, as nearly aseptic as can be, that will close these openings well into, but not through, the apex, will insure apices infected as little, and filled as much, as possible, and functional teeth.''

There is little use in consuming time with teeth with septic pulps, for the reason that no adequate treatment is known which will certainly cure suppurative, granulating, or cystic phases of apical disease except surgery. This statement implies recourse to (*a*) apicoectomy, which is rarely successful, particularly on the multi-rooted teeth, because the apical section of dentin and cementum left by the operation is not sterile, and generally if such tissues are sterile, apical disease does not exist, hence there is no need of apicoectomy; or, (*b*) extraction, which obviously destroys the tooth, but does not cure the bone lesions, except when followed by (*c*) curetment, the third and most important surgical expedient.

Let us consider the second portal of entrance, the gingival crevice. Dewey and Noyes have demonstrated the association of three sets of lymph vessels, with blood supply, that completely surround every tooth, which is seated, so to speak, in a basket of periodontal membrane, dense socket lining (*lamina dura*) and blood-vessels, all of which tissues these lymph vessels penetrate, nourish and drain. The largest group passes from the gingivae centrally down through the membrane, paralleling the cementum; it is joined by gingival and middle-third groups, the first of which carries infection from the gingival crests to the nearest lymph nodes. Thus, a superficial gingival infection would be deflected over the interseptal crests; but a periodontal infection would finally reach the subapical tissues, splitting the periodontal membrane on its way, as in a true pyorrhea pocket.

So great has been the divergence of opinion regarding the periapically involved tooth that the dental profession, excepting the few who are trying to follow the saner middle course of judgment based on a foundation of pathology, comparing the clinical results with the laboratory findings, making a thoughtful study of the history, and combining with this the experience of observation, is divided between the (1) *radical*, who insists that all pulpless teeth are a menace, and must be removed, and the equally dangerous (2) *conservative*, who, not having noted the consistent improvement in the general health of the patient after extraction, discounts the idea of oral lesions being possible foci, and holds that all pulpless teeth should be treated and retained, that the masticatory apparatus be not impaired.

Concerning the radical or 100 per cent. vitality man, have we not reached a period when it would be well to pause and seriously reflect upon the consequences, the favorable and the disappointing results of radical tooth extraction, when such an operation is presented as a panacea for *all* bodily ills? Many of us know of cases wherein the dentist has advocated or the physician has demanded complete eradication of not only proved focal infectious areas, but *all* suspected areas on the ground of their potentiality for evil.

For the last five years, although today very much modified, a hysteric wave has swept the ranks of both the medical and the dental professions, swamping them, in many instances, with the idea that tooth-extraction was the great cure-all. Too well do many of us know of the thousands and thousands of valuable teeth, teeth that we many times were confident had not been in any way contributing agents to the systemic symptoms, ruthlessly sacrificed upon the altar of this strange god.

Is not the time at hand when the thinking men of dentistry should persistently call a halt upon this destruction without reason? It is natural that we should think of dental infections and of the part they play as etiologic factors in systemic disease. No one that has seen the astonishing results experienced by some patients after the elimination of dental foci will deny that the theory contains a reasonable amount of truth. I think we all admit this, even though we know that many cases are not apparently benefited by such treatment, and even though we feel that much remains to be explained before this theory can be accepted as a scientific fact.

There seems to be a crying need for a broader range of vision and a better balance to meet the present situation. We then will be in a position to offer our patients a greater protection from such experimentation. The agitation of the subject of focal infection and the concentration on the teeth as one of the chief sources of this infection has been responsible for this wave, which has created such havoc among the ranks of the two great branches of the healing art and left them running in circles.

Repeatedly have words of warning been spoken by thoughtful men against this radical procedure, which has been practiced under the guise of scientific treatment, and while, as I have stated, there has come a modification, still in many instances there continues a shameful sacrifice. To minimize the evil effects of neglected oral conditions is not my intention, for no one of conscience would argue for the retention in the mouth of diseased teeth which cannot be cured, but the wholesale slaughter of harmless teeth or teeth that can be made harmless by treatment, which has been going on in the recent past, does constitute in itself one of the most serious reflections that has ever been cast upon the profession.

"Infection about the mouth probably stands first in point of frequency as a cause of metastatic infection," says Irons, who generously adds, "but the entire burden of effort for the recovery of the patient should not be arbitrarily thrown on the dentist." This expresses the sentiment of your essayist, who feels that the dental specialists can only be of real aid to the physician when the physician works in co-operation with and correlates the post-operative findings with those of the dentist. In this way the problems of each will be understood by the other, and the patient will receive the more intelligent service.

We should clearly draw the line between the atrium of infection and the focus of infection. An atrium of infection is a point of entrance for infection and may be normal tissue, while a focus of infection, Dr. Billings tells us, is "a circumscribed area of tissue containing pathogenic micro-organisms." It is therefore possible for infection to arise from bacterial absorption from a tissue that is chronically harboring bacteria, or it may result from bacterial penetration through normal tissue.

General disease, which is caused either directly or indirectly by dental foci, cannot be discussed without considering the so-called "dead tooth." Perhaps no term in dentistry has been so poorly defined, so overworked and so often incorrectly applied, as "dead tooth"; as a consequence much confusion exists regarding the correct classification of this term. The pulpless tooth is not always a "dead" tooth as is commonly believed, for since the nutrition of some pulpless teeth may still be maintained by the lymphatics of the alveolar process functioning through the still vital lymphatics of the unbroken periodontal membrane, such a tooth does not necessarily have to be condemned because it fails to transmit sensation. Pulp death does not fatally impair tooth nutrition; a tooth in this condition is more susceptible, we agree, and must be kept under survey, but on this account solely should not be marked for extraction.

Current discussion in dental circles regarding the "dead tooth" is quite heated. Grieves clearly describes the situation when he says:

"There are so many bodily ills of doubtful etiology, hence treated experimentally, which are said to have been caused by non-vital teeth and cured by the removal of such teeth from the maxillae; so doubtful pathologically are these ills, so questionable to the claim "cured," when applied to the chronic systemic or the equally chronic oral disease, that these and other reasons have prompted a pause by the medical and the dental profession, in order to classify and define the pathology, not only of pulpless teeth, but all other teeth, invaded by pathogenic bacteria."

This has been painstakingly worked out by him in the "Classification of Teeth with Diseased Pulps," wherein he presents a classification and a terminology, which is of great assistance to the physician and the dentist in arriving at a point where a mutual understanding of the different classes will obtain.

It is not the wish of the writer to convey the idea that he believes the majority of cases of general infection have as a contributing factor some oral condition, nor that diseased teeth in all cases are primarily the cause of systemic symptoms; he does, however, wish you to consider the importance of the fact that there have followed in many cases most gratifying results as a sequence when a thorough and intelligent oral diagnosis, followed by such dental treatments or oral operations as were indicated, were included in the treatment.

Dr. DeSchweinitz has aptly expressed the idea of co-operation between the physician and the dentist, when he said:

"We must not act along lines of ill-considered and reckless sacrifice, but with due care and judgment, weighing the evidence as it presents itself from the investigation of all sources from which the etiological factor may spring, and not from one alone.

Before concluding let me urge that a greater effort be made by the dentist to so apply and equip himself that he will enjoy a broader knowledge of the underlying principles which we today are practicing, thereby appreciating more fully the *true importance* of our *responsibility* in making a *correct* oral diagnosis, and by this knowledge be able to apply more scientifically such treatment as will be indicated to the end that our continued investigations will ultimately lead us to determine the specific relation of infectious lesions in the mouth to general health.—*Dental Cosmos.*

DISCUSSION

*Dr. C. H. Frink*—Mr. President, Dr. Brun and Gentlemen: This paper seems to be really a most worthy companion paper to the most excellent one we heard from Dr. B. Holly Smith, Jr., yesterday. I wish to emphasize one point brought out in the essayist's paper,—that of the necessity and importance of a more cordial relation between the physician and dentist with reference to diagnosis and treatment of oral diseases or disturbances. All of you, I know, can recount numerous instances in your own practices such as happened when I was called to a hospital by a physician friend of mine recently. The diagnosis had already been made that the patient had two impacted cuspids, and I was informed that the systemic trouble had been traced definitely to this condition and that they must be removed, therefore I was asked to bring my instruments and the patient would be prepared. Upon reaching the hospital I found the patient under ether and the roentgenograms, hurriedly examined, showed two cuspids but not impacted, simply the normal picture of unerupted cuspids with accompanying normal rarefied area between the permanent cuspids and the absorbing shells. The patient being eleven years of age would instantly preclude the possibility of impactions. I did not know what to do. If I had known beforehand I would not have done anything. Under the circumstances, I extracted the two deciduous teeth and did a little curetment, excusing myself with the thought that perhaps an infected area existed. This was one of those peculiar cases, and strange to say the patient got well, but I am loath to believe it was from the extraction of these two deciduous teeth.

The essayist spoke of so many men reaching hasty conclusions from the apparently successful treatment of one or two cases and jumping into print to announce some wonderful cure, only to find it fail when subsequently tried. This brought to my mind what was said by some practitioner of medicine—I think it was a London man—who after fifty years of practice, when asked to define medicine, replied, ''Medicine is the art of conjecture and the science of guessing.'' And so, as we grow older in practice, things are brought home to show that the healing art is not so exact as one might think.

The essayist made the remark that ''apicoectomy is rarely successful.'' I agree with him and I simply say that we undoubtedly made a fad of apicoectomy. We have been faddists all along. We made a fad of gold crowns and bridge work, extension for prevention and other theories. And what a fad we made of cataphoresis. At the meetings of the Southern and American Dental Associations at Old Point Comfort, Va., (when the National Dental Association was organized) every clinic I witnessed was on cataphoresis. Then in rapid succession, soft gold, porcelain inlays, silicate cements and analgesia all contributed to the odontological craving for faddism. A fad is a popular innovation, a capricious hobby, a passing fancy, or it may be a useful technique or process discriminately and indiscriminately used; so, I am only condemning the indiscriminate use. Now in the past few years extraction has been the fad, and what we were wont to style ''dental parlors'' can well be given the cognomen of ''drawing rooms.''

*Dr. Edwin J. McQuillan*—Mr. President, Dr. Brun and Gentlemen: I listened with much pleasure to Dr. Brun's paper and I feel he covered the subject very well. One point well taken is that the dentist should brush up on pathology. Today there are several books published and on the market which combine a general treatise on dental and medical pathology together. We come in contact with physicians day after day and half the time we do not know what they are talking about. In every new field, whether it is dentistry or anything else, there are a certain number of leaders and a great number of followers, and I do not think myself that a man should think because he reads articles in the dental journals written by some leading men that that covers the whole story. They take the stand in some sections, and I think some quote Dr. Grieves as an authority for it, that wherever they find a general systemic condition, and the possibility of a tooth being a factor in that condition, to remove the tooth. There is much contention today about the curetment of sockets, but every man that does this work should map out a plan for himself and not be governed by what somebody else has done. Recently I saw an article in one of the journals saying that the Mayo Brothers will not take a case for treatment where there is a dental condition showing in the radiogram and the patient insists on retaining those teeth in the mouth. If they do insist, they will not treat them at all. Another thing to consider for the exodontist is that he is having cases referred to him and he does not really know what stand to take. Recently I had a case sent to me by a man who for the last twenty-five years has been in general practice. It was a case of a lower right molar that had been treated years ago, and the radiogram showed a pronounced granuloma on both roots. He had opened up that tooth, treated it and made a beautiful gold cap, and then he referred the patient to me to curet the abscess. There was a condition presented to me where it was just a case of go back and fight it out with this man. I think, myself, that there is a wonderful field for this work.

*Dr. Geo. Milholland*—Mr. President, Dr. Brun and Gentlemen: There is one point that Dr. Brun brought out very well, and that is immediately rushing into print after we have found a cure effected by the extraction of abscessed teeth, with this as a panacea for the relief of all systemic troubles. He has wisely said the thing to do, is first to find out whether the percentage of cases are cured by the removal of diseased teeth before we put ourselves on record. In my practice it has been my custom, for cases sent me by physicians for examination of the mouth for a possible foci of infection, first to inquire if a thorough examination has been made of all the sinuses and tonsils, and also to inquire of the physician if a Wassermann test has been made. Most of the cases of locomotor ataxia we attribute to syphilis. After being positive the above examinations have been made, then it is our duty to have the teeth radiographed, and remove only those teeth that are infected. I know of a case of a man about fifty years, who was apparently in good health, with the exception of pains in his back which at times were so severe that he was finally compelled to quit work. He had been treated for rheumatism, neuritis, etc., with no result. Finally his mouth was examined. He had nearly all his teeth, and they were in fairly good condition, with the exception that they were worn down a great deal from the excessive use of chewing tobacco. It was decided they all should be removed. He was taken to the hospital, given ether, and they were removed. In extracting the right superior bicuspids there was quite a flow of pus from the sockets, which indicated that there was antrum trouble. His antrum afterward was treated, and a speedy recovery resulted. Now the question arises, were the teeth the cause of these pains or was the antrum? I relate this case simply to show that we should be a little more conservative and not extract until we are definitely sure that every other possible infection has been eliminated.

*Dr. Brun* (closing the discussion)—In closing I would like to emphasize one point that Dr. Frink has spoken of as to the relation between the physician and the dentist. To my mind this is a most important thought, and in this relation much depends upon the dentist. We have all worked hard to place dentistry upon a higher plane. To-day dentistry, as we all concede, is no longer solely the application of higher mechanical principles. There is something else—a bigger factor. If we wish the respect of the physicians, we must have a knowledge that will allow us to intelligently report to them, that they may intelligently apply our findings. In many instances the reports made are so negative that they do not give the physician that which he desires; and so, in this respect, I feel that we are standing in our own light. If a better knowledge of histo-pathology is obtained by the dentist, then he will be better able to present the dental side of the case to the physician.

Dr. Frink spoke of the art of guessing regarding medicine. We must all guess at times, but let us have some reason for our conclusions. Two of the biggest surgeons in Baltimore were conferring over a case. One man said, "Doctor, what do you think the condition is?" The other replied, "I am sure that he has so and so." The next question was, "Why do you say that? My opinion is the contrary." The answer was, "Well, I cannot say *why*, but I will wager that my diagnosis is correct." To which the first replied, "I tell you I would rather be wrong with a reason, than be right and not know why."

Many of us try and do all that is demanded of us—we do a general anesthesia with one hand and a tooth extraction with the other. There was a time when this was the accepted practice, but to the thinking dentist of today, such methods are no longer considered. The dentist should not be so free in the administration of general anesthetics in his office, unless he is in a position to take care of his patient properly both from a pre- and post-operative standpoint, unless he is intimately familiar with the anesthetic agent, and unless he is surrounded by skilled assistants and proper equipment. My idea is that any anesthetic that is sufficiently powerful to produce sleep is sufficiently dangerous to require the administrator's undivided attention. While our fathers were the pioneers, and through ignorance did many things in their day that we should not consider for a moment today, is no excuse for our making the same mistakes, because of tradition, when use and research have proved the error of their methods.

In the case of the antrum spoken of, if the dentist had been more careful in his examination, had given more attention to the history of the case and considered his responsibility when making the examination for the diagnosis, he would have determined whether or not the sinus infection was due primarily to the bicuspid or was due primarily to other causes. In a case of this classification the dentist should not confine himself solely to questioning the physician, but should have such knowledge as to allow him to make a correct diagnosis and be able to answer any questions the physicians might ask. When the case is purely of dental origin, the dental man is the logical one to make the diagnosis, and when patients are referred to us for an opinion we should have equipped ourselves with such knowledge as will allow us to make a complete diagnosis and report. I thank you very much for your attention.—*Dental Cosmos.*

